# Economic burden of out-of-pocket expenditure, productivity cost during pregnancy and COVID-19 impact on household economy in a cohort of pregnant women in Anuradhapura District, Sri Lanka; A study protocol

**DOI:** 10.12688/f1000research.53320.1

**Published:** 2021-07-30

**Authors:** Sajaan Praveena Gunarathne, Nuwan Darshana Wickramasinghe, Thilini Chanchala Agampodi, Indika Ruwan Prasanna, Suneth Buddhika Agampodi

**Affiliations:** 1 Department of Community Medicine, Faculty of Medicine and Allied Sciences, Rajarata University of Sri Lanka, Anuradhapura, North Central Province, 50000, Sri Lanka; 2 Department of Economics, Faculty of Social Sciences and Humanities, Rajarata University of Sri Lanka, Anuradhapura, North Central Province, 50000, Sri Lanka

**Keywords:** COVID-19 impact, out-of-pocket expenditure, pregnancy, productivity cost, Sri Lanka

## Abstract

**Background: **Investigating the out-of-pocket expenditure (OOPE) associated with maternal health is important since OOPE directly affects the affordability of health services. Global evidence suggests the importance of capturing the productivity cost during pregnancy in terms of absenteeism and presenteeism. Furthermore, the impact of the ongoing COVID-19 pandemic on the household economy needs to be further evaluated as pregnant women are one of the most vulnerable groups. This study aims at determining the economic burden of OOPE, productivity cost, and COVID-19 impact on pregnant women's household economy in a cohort of pregnant women in Anuradhapura District, Sri Lanka.

**Methods: **The study setting is all 22 Medical Officer of Health (MOH) areas in Anuradhapura district, Sri Lanka. The study has three components; a follow-up study of a cohort of pregnant women to assess the magnitude and associated factors of OOPE and to assess the productivity cost (Component 1), a qualitative case study to explore the impact and causes of the OOPE under free health services (Component 2) and a cross-sectional study to describe the effects of COVID-19 outbreak on household economy (Component 3). The study samples consist of 1,393 and 1,460 participants for components one and three, respectively, and 25 pregnant women will be recruited for component two. The data will be analyzed using descriptive, parametric, and non-parametric statistics for the first and third components and thematic analysis for the second component.

**Discussion: **With the lack of evidence on OOPE, productivity loss/cost in terms of maternal health, and COVID-19 impact on household economy in Sri Lanka, the evidence generated from this study would be valuable for policymakers, health care administrators, and health care practitioners globally, regionally, and locally to plan for future measures for reducing the OOPE, productivity loss/cost, and minimizing the economic hardship of the COVID-19 outbreak during pregnancy.

## Introduction

The concepts of out-of-pocket expenditure (OOPE) and productivity cost derived from the cost of illness studies in health economics describe the total health economic cost in terms of direct, indirect, and intangible categories. ^1– 5^ Globally, estimating an illness’ total financial cost in direct or indirect dimensions is widely used to aid in evidence-informed policymaking. ^4^

The direct cost can be defined as all the monetary expenses or the OOPE due to a disease or any health concern. ^2^ The World Bank interprets the OOPE as ‘any direct payout by households, including gratuities and in-kind payments, to health care providers of pharmaceuticals, therapeutic appliances, and other goods and services whose primary intent is to contribute to the restoration or enhancement of the health status of individuals or population groups’. ^6, 7^ The indirect cost refers to the productivity losses related to morbidity and mortality, borne by the individual, family, society, or the employer. ^1, 3^ The term ‘productivity cost’ or ‘productivity loss’ can be defined as reducing efficiency or output in terms of income-generating or day-to-day activities. ^8^

In the global health agenda, the OOPE is considered a vital issue due to its negative impact on achieving the Sustainable Development Goals (SDG); especially goal three focusing on ‘ensuring healthy lives and promoting wellbeing for all ages’. ^9^ Also, there are individual, family, society, and national-level negative consequences of the OOPE such as leading people in to poverty, debt burden and unbearable household expenditure, misusing the health care resources, barriers to accessing health facilities, and barriers to achieving the free health care policy targets. ^10^ Productivity loss, on the other hand, creates a high economic burden and has been repeatedly found that adverse health conditions increase work-related absences (absenteeism) and decrease productivity while at work (presenteeism), creating a substantial economic burden on industry as well as on household activities. ^11– 16^

The OOPE and productivity cost influence are further highlighted in essential health care sub-sectors, such as maternal health services provision. ^17– 19^ To build a healthy nation, the focus is given to stimulate and protect maternal health, and there are many health services focused on maternal health. ^10, 20^ Improvement in maternal morbidity and mortality indicators critically depends on the availability, accessibility, and affordability of reproductive health services. Global evidence suggests that the significant barrier for that is enormous OOPE. ^21– 24^ It is reported that the accessibility and the affordability of maternal health facilities are negatively affected by the OOPE. ^25^ Besides, evidence suggests that productivity loss/cost is higher during pregnancy due to maternal ill-health conditions, ^19^ affecting income-generating activities and routine household works. ^11– 16^

In addition, the COVID-19 outbreak, which is an unexpected change in the global and national landscape on daily wellbeing, ^26, 27^ affects both social and economic, affecting households directly or indirectly. ^26^ Few significant concerns are that the pandemic affects the household economy and disrupts health-service delivery, including newborn and maternal health services, particularly in resource-limited and lower-income countries. ^27^ Unavailability of accessible or affordable health services during the pandemic may create low accessibility for pregnant women in lower-income groups. Few studies and reports cited the adverse impact of the COVID-19 outbreak on the household economy ^28, 29^; however, the studies focusing primarily on household economies of pregnant women are lacking. However, the impact of the COVID-19 outbreak on maternal health service utilization needs to be further studied since the World Health Organization (WHO) has highlighted pregnant women among the vulnerable groups of COVID-19, particularly in low-income countries. ^30^

Currently, Sri Lanka has well developed maternal health care package in both public (in a larger extent) and private health system which is accessed by almost all pregnant women. ^31^ Considering the SDG in terms of maternal and child health targets, Sri Lanka is among the leading countries of the lower-middle-income countries. The government provides free public health services on maternal care with a well-developed network of primary health care officers and reports almost full coverage in antenatal care in free public health system. ^32^ Even in the outbreak of COVID-19 infection, the interim guidelines on maternal and child health services were provided to improve maternal health during the pandemic by health care authorities in Sri Lanka, ^29^ which balances health service utilization for all income categories.

However, evidence on the OOPE, productivity loss, and COVID-19 impact on pregnant women in Sri Lanka are lacking. A detailed estimation of the OOPE and productivity loss in maternal care and assessing the impact of COVID-19 outbreak on health service utilization of pregnant women and their household economy would be important in generating evidence on the manifestations of OOPE and productivity loss and decision making during pandemic situations in a country with high maternal health service standards.

The study will focus on,

I. Estimating the magnitude of OOPE and assessing the productivity cost during pregnancy
II. Determining the associated factors of OOPE
III. Exploring the reasons for the OOPE and the effects of OOPE on the household economy of pregnant women
IV. Assessing the COVID-19 impact on pregnant women’s household economy in a rural Sri Lankan setting

## Methods

The study protocol is prepared according to the consolidated criteria for reporting qualitative research (COREQ)/Standards for reporting qualitative research (SRQR) guidelines.

### Study setting

This study will be conducted in Anuradhapura, situated in the North Central Province and the largest District in Sri Lanka (7,179 km ^2^). The reported total population is 902,930. The majority (92.7%) lives in rural areas and the leading ethnic group is Sinhalese (90.7%). ^34^ The median household income recorded as LKR 41,629.00 (USD 285.91) per month and the primary source of income is Agriculture. ^33^ In the area, the health expenditure documented as 42% from the private sector, 22% from the government, and 36% from other government sources. ^35, 36^

**Figure 1.**
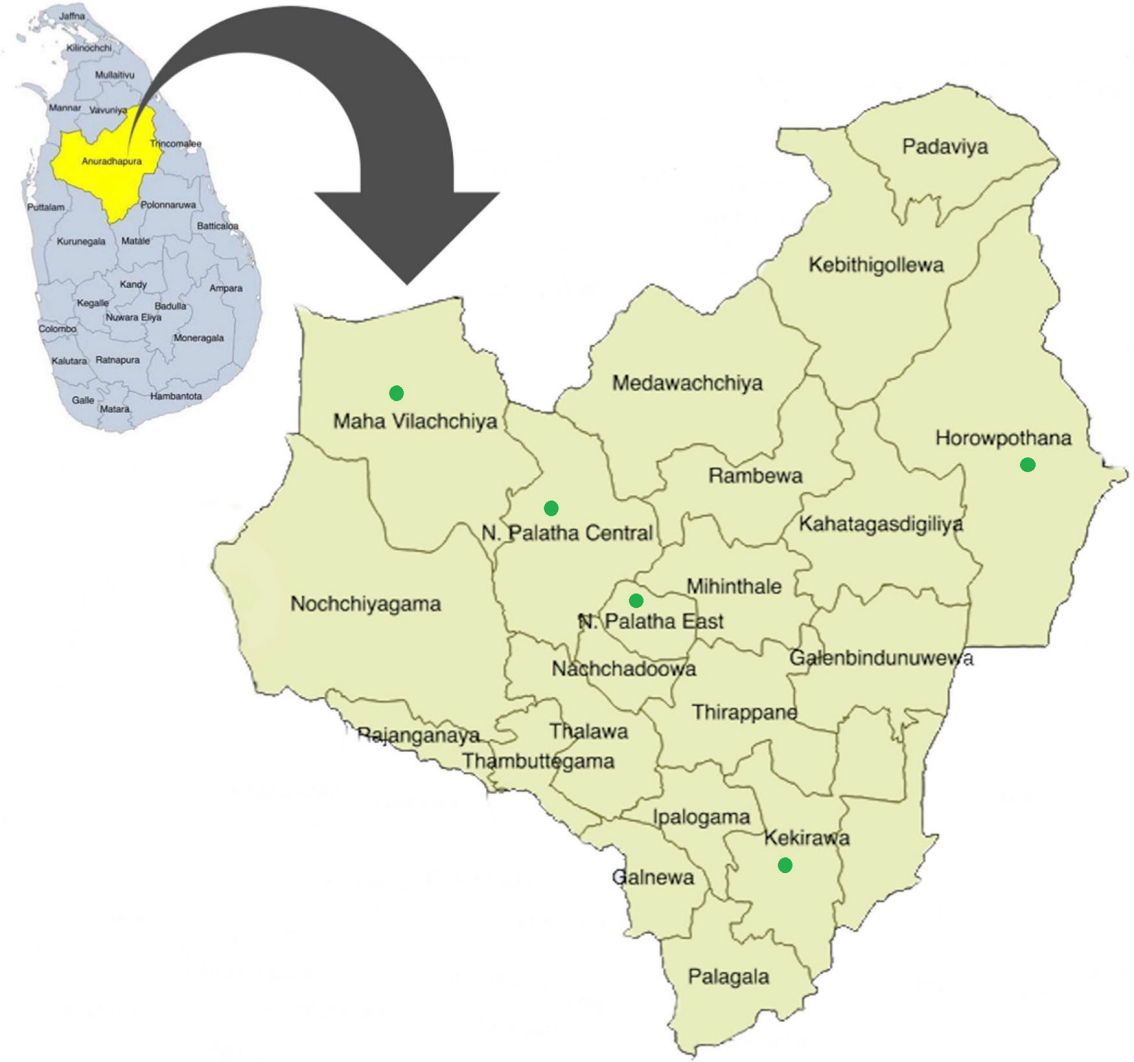
Spatial distribution of 22 participant recruitment areas of RaPCo study in the Anuradhapura district. (The figure has been reproduced and edited with the permission from Agampodi TC, Wickramasinghe ND, Prasanna RIR, Irangani MKL, Banda JMS, Jayathilake PMB, *et al.* The Rajarata Pregnancy Cohort (RaPCo): study protocol. BMC Pregnancy Childbirth [Internet]. 2020 Jun 26 [cited 2020 Nov 30]; 20(1):374. Available from https://bmcpregnancychildbirth.biomedcentral.com/articles/10.1186/s12884-020-03056-x)

The maternal health services for pregnant women are provided through the Medical Officer of Health (MOH), and there are 22 MOH areas in the Anuradhapura district ( Figure 1). MOH is the health administrator at the divisional level and conducts activities through a subordinate network of public health officers. In addition, the district consists of 275 public health midwife (PHM) areas, and each PHM area has 1,500-4,000 of population. PHM provides domiciliary and clinic care for antenatal mothers and children less than five years of age and therefore identifies as the grass root level primary health care provider. Each woman who is eligible to be pregnant is registered under the PHM. ^37^ Approximately 17,000 expectant women were reported in the area in 2015. The public health system exerts 96% coverage in antenatal care, ^38^ and the number of live births was 15,376. ^39^ The study protocol of the Rajarata Pregnancy Cohort (RaPCo) has already been published and includes further information on the study setting, including MOH and PHM areas. ^39^

### Study design

**Figure 2.**
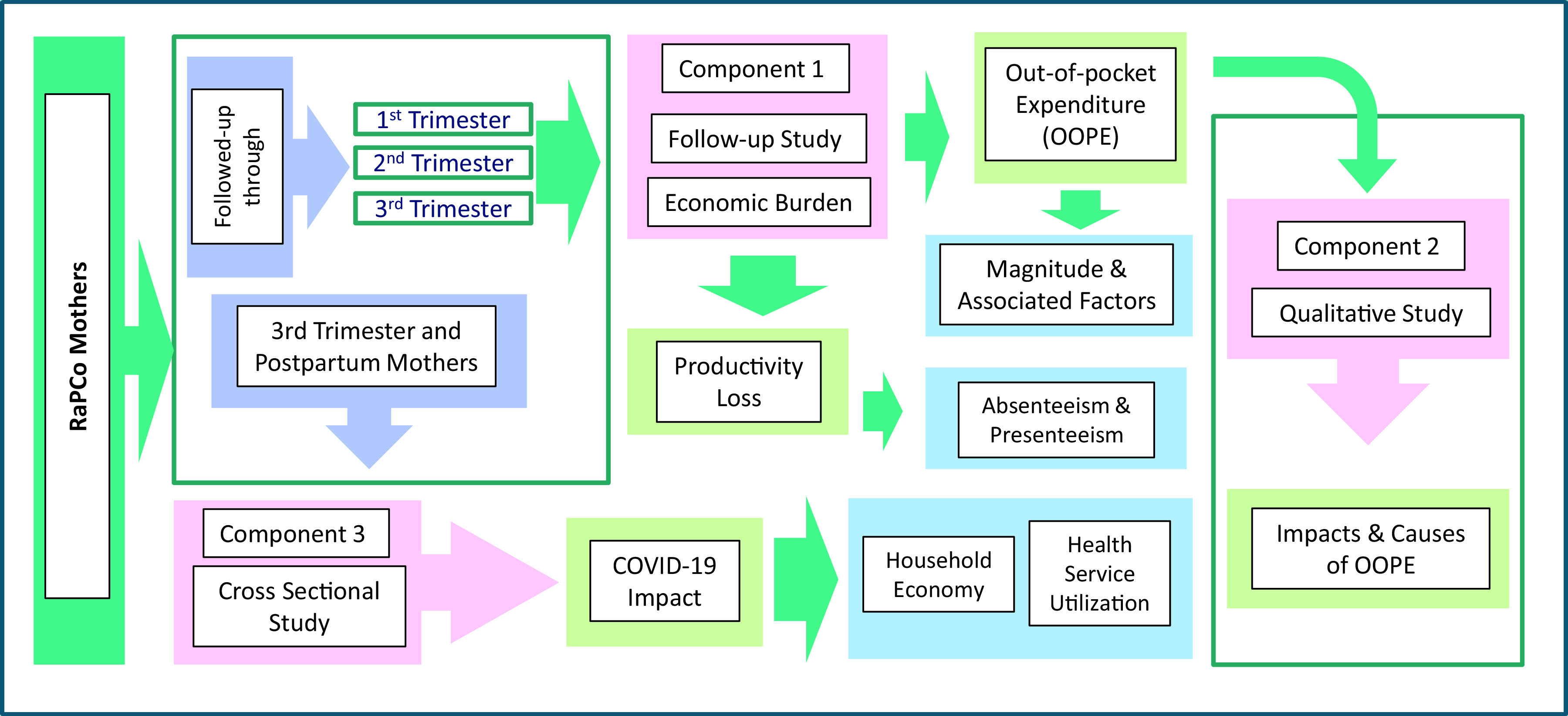
Study design.

The present study is a part of an extensive cohort survey of pregnant women residing in the Anuradhapura district, Sri Lanka, namely the Rajarata Pregnancy Cohort (RaPCo) and the protocol of the RaPCo study was already published. ^39^ This part of the research has three major components; a follow-up study of a cohort of pregnant women to find the magnitude and associated factors of the OOPE and assessing the productivity loss/cost (Component 1), a qualitative case study based design to explore the reasons for the OOPE and the effects of OOPE on the household economy of pregnant women (Component 2) and a cross-sectional study to find the effects of COVID-19 outbreak on the household economy of pregnant women (Component 3). The study design is presented in Figure 2.

### Study population

The study population for this study included all pregnant women residing in the Anuradhapura district, Sri Lanka.

From here onwards, the methods are presented based on the three components of the study.

## Component 1: Finding the magnitude and associated factors of the OOPE and assessing the productivity loss/cost during pregnancy

### Study sample/participants

The RaPCo study’s population (3,400 pregnant women) included all pregnant women living in the Anuradhapura district from July to September 2019 according to the following eligibility criteria. ^39^

Inclusion criteria:

1. Pregnant women who were registered in the “pregnant mothers’ register” of public health midwives (PHMM) and attending antenatal clinics in the Anuradhapura district
2. Pregnant women who hold a permanent residence in the Anuradhapura district for the year ahead
3. Pregnant women with the period of amenorrhea (POA)/gestational age (GA) less than 13 weeks at the recruitment

Exclusion criteria:

1. Pregnant women who have uncertain due dates
2. Pregnant women who have planned to leave the study setting for delivery

Although the study was initially planned to recruit the total cohort at the baseline, the participation was volunteer to provide such information considering the sensitive nature of data that included the in-depth household and health-related financial details. Therefore, a sub-sample of RaPCo was selected, and 1,393 pregnant women were willingly attended to provide their financial details.

### Participant recruitment

Participant recruitment has already been completed. All PHHM were asked to send an invitation for all pregnant women according to the eligibility criteria and a special clinic was done fortnightly at each MOH to recruit study participants from July to October 2019. Written informed consent was obtained from all the study participants before the recruitment and explained the participation is volunteer. Ethical clearance was taken from the Ethics Review Committee, Faculty of Medicine and Allied Sciences, Rajarata University of Sri Lanka.

### Data collection

During this follow-up study, data collection was done in four stages to cover the three trimesters and hospitalization for delivery. The study tools were self-administered questionnaires and interviewer-administered questionnaires. All self-administered questionnaires were added to an information leaflet about the questionnaire and guidelines to fill in, and the study’s overall objective. All the study instruments were pre-tested and modified after the pre-testing. The pre-testing was conducted at the Primary Medical Care unit, Puliyankulama, and the MOH office of Mahawilachchiya, Anuradhapura District, Sri Lanka.

Trained data collectors under pre-intern medical officers’ supervision were employed for each MOH, and 22 data collectors participated. The Principal Investigator (PI) trained all data collectors and checked the status regularly. Also, phone reminders were carried out to hold pregnant women’s responses for the study period.

*Baseline survey*

Baseline data collection was conducted during the 1st trimester (within the first 12 weeks of pregnancy). An interviewer-administered questionnaire was employed to gather the socio-demographic data of pregnant women, and a self-administered questionnaire was used to collect,

• Household income (by occupation and by other sources)
• Household expenditure details [medical, non-medical (food, transport, education, utility, and others)]
• Financial sources of health care expenditure
• Details on OOPE (identification of pregnancy, cost related to the booking visit, cost associated with seeking medical care)
• Breakdown of OOPE in terms of direct medical expenditure (cost for laboratory investigations, consultation, medicines, and hospitalization charges) and direct non-medical expense (for traveling, foods, and refreshments, accompanying persons, and others)
• Details on productivity cost/loss (absenteeism and presenteeism with and without compensation for coping strategies)

The average duration for the baseline survey under the interviewer-administered questionnaire was 20-40 minutes. All pregnant women were asked to fill the self-administered questionnaire conveniently at home and hand over the filled version to the MOH office within two weeks.

*First follow-up survey*

The first follow-up survey was conducted in the second trimester (from the 13 ^th^ to 26 ^th^ week of the pregnancy period). Three copies of a self-administered questionnaire were distributed to be filled in by the participants at home to collect the health expenditure (OOPE) data and productivity loss monthly. The information regarding the date and time of the first follow-up survey was given to women via PHMs and phone reminders. The questionnaires were distributed to all participants at the first follow-up survey was conducted in the MOH offices in the Anuradhapura district.

*Second follow-up survey*

In the second follow-up, three copies of a self-administered questionnaire were distributed to be filled in by the participants at home to collect monthly health expenditure data and productivity loss during the third trimester (from the 27 ^th^ week onwards). The information regarding the date and the time of the second follow-up survey was given to the participating pregnant women via PHMs and phone reminders. The questionnaires were distributed to all participants at the second follow-up survey conducted in the MOH offices in the Anuradhapura district.

*Hospital survey*

The hospital survey was conducted to collect the associated cost for delivery in the hospital or any other health institute using an interviewer-administered questionnaire. All pregnant women in the study sample, and their relatives were instructed to inform PI when hospitalizing for delivery. The data collection was done at the hospital with the permission of health authorities by pre-intern medical officers. The average duration of the interview was 10-15 minutes, and participants were informed that participation is voluntary.

### Data management

Data collected from the interviewer-administered questionnaire of the baseline survey and the hospital survey was entered directly electronically into an online platform and converted to a Microsoft Office Excel (MS Excel) datasheet for data cleaning. The rest of the data from the self-administered questionnaire was entered by a single entry technique into MS Excel datasheet by three research assistants (RA) and PI, and the PI manually verified 10% of the data entered by RA. Data cleaning was done using MS Excel and transferred to Statistical Package for Social Sciences V27 (SPSS V27) for data analysis. After the data entering, all the questionnaires were stored in a restricted place and, separated from the participants’ consent forms and any identification details.

The monetary values of all the household income, expenditure and OOPE related data which were collected in Sri Lankan Rupees (LKR) were converted to United States Dollar (USD). The average exchange rate of USD to LKR in July 2019 (USD 1=LKR 176.38) was used for converting. ^40^

### Plan for data analysis

Data will be analyzed using the SPSS V27. Three aspects will be considered under the component one; assessing the magnitude of OOPE, assessing the productivity cost, and assessing the associated factors of OOPE.

*Assessing the magnitude of OOPE*

The magnitude of OOPE will be computed based on the sum of;

• Medical expenditure (for laboratory investigations, consultation, medicines, and hospitalization charges)
• Non-medical expenditure (for traveling, foods, and refreshments, accompanying persons, and others)

Descriptive statistics will be used to obtain the summary measures of the OOPE; mean (standard deviation) [mean (SD)], median (interquartile range) [median (IQR)].

According to three trimesters (OOPE per month in each trimester), these calculations will be carried out, and the sum of three trimesters will be considered the total OOPE during pregnancy. The statistical significance of the difference between the OOPE during each trimester will be tested using the Repeated Measures Analysis of Variance (Repeated Measures ANOVA) technique.

Further estimation of OOPE will be carried out based on different dimensions.

• The magnitude of OOPE per month/trimester and for throughout the pregnancy
• The proportion of OOPE from total household expenditure and income
• OOPE for free health services and paid health services
• OOPE for routine medical care and specialized medical care
• OOPE for complicated pregnancy/maternal morbidities

*Assessing the productivity cost*

The productivity cost will be calculated based on the sum of;

• The time when the participants were utterly unable to work due to illness (absenteeism)
• The time when they worked with reduced efficiency while they were ill (presenteeism)

Further, coping strategies will be recognized due to the information collected concerning the assistance received by pregnant women for routine activities during the episodes of ill health. This will be calculated as intra-household adaptation (the help of household members to carry out daily activities), adaptation involving social networks (using a relative/friend/neighbor to carry out day-to-day activities), and hired support during the episodes of ill health. Adjusted productivity loss will be calculated after deducting this measure from the sum of the total productivity loss (absenteeism and presenteeism), compensated for coping mechanisms.

Productivity loss will be translated into monetary terms to calculate the production cost, and it will be based on the mean daily per capita income. The per capita income will be calculated based on equal weight given to all household members engaged in income-generating activities and assuming that work carried out by pregnant women is equally significant in contributing to the household income.

Descriptive statistics will be used to summarize the productivity loss, productivity cost (with and without compensation for coping mechanisms), mean (SD), and median (IQR).

All these calculations will be carried out according to three trimesters, and the sum of three trimesters will be considered the total productivity loss/productivity cost. The statistical significance of the difference between the trimesters will be tested by Repeated Measures ANOVA techniques.

*Assessing the associated factors of OOPE*

Parametric or non-parametric procedures (after checking normality) will be performed to identify the statistical significance. The Multiple Linear Regression (MLRM) technique will be carried out to find the impact of associated factors of OOPE.

The dependent variable for the model will be the OOPE, which is a continuous variable, and the independent variables will include binary, categorical (which will consist of as dummy variables) and continuous variables; household average monthly income, the household average monthly cost, age of the pregnant women, ethnicity, religion, educational level, the status of health education, household size, condition of the house, preferred health care mode, transport mode, occupation, occupation of the household head, number of pregnancies, having a maternal ill condition, maternal comorbidities and MOH Area.

The regression model’s outcome (estimators) will be tested using the t-test (under 0.01, 0.05, and 0.1 significance levels). The residual analysis will also be conducted to confirm the accuracy and whether the model has violated the assumptions. Further, F-statistics will be used to identify whether the model has a better overall significance, and both R-square and adjusted R-square techniques will be used to check the goodness of fit of the model.

## Component 2: Exploring the reasons for the OOPE and the effects of OOPE on the household economy of pregnant women

### Study sample/participants

This qualitative component will include in-depth interviews with pregnant women attending antenatal clinics who use free health services (routine antenatal clinics, government hospitals, and health care centers) and focus group discussions (FGDs) with the PHMs. PHM is the most suitable person outside the home to collect information regarding the reasons for pregnancy-related expenditures. The participants will be recruited to represent urban, rural, and minority ethnic communities ^37^ in the District ( Table 1) and presented in green color points in Figure 1.

**Table 1.** The selected study areas for different communities.

Community	Selected area
Urban and semi-urban community	• Nuwaragam palatha East (NPE) • Kekirawa
Rural community	• Nuwaragam palatha central (NPC) • Vilachchiya
Minority ethnic community	• Horowpothana

## Participant recruitment

Recruitment will be done at the selected five antenatal clinics at MOH offices to cover the urban, semi-urban, and rural and minority ethnic communities. Five participants from each antenatal clinic will be purposefully selected based on the service utilization. Pregnant women attending the government field clinics regularly will be considered as “using government services.” We will classify and purposefully select subgroups of pregnant women who will use mainly the government public health sector for antenatal care services. These subgroups will be selected based on that they have never attended or have attended the private sector only once or twice during the antenatal period to either consult a Consultant Obstetrician or get the routine Ultrasound Scan done. Although five participants from each antenatal clinic will be initially surveyed, additional participants will be interviewed until the data saturation point, compiling enough information to understand the phenomena under the investigation. PHMs willing to participate in FGDs will be recruited for each MOH area. Five focus group discussions with PHMs will be conducted initially to resemble semi-urban, rural, and minority ethnic communities. The point of data saturation will determine the number of FGDs. The study participants, pregnant women, and PHMs, will be notified of the study aims, and written informed consent will be sorted before the interviews.

## Data collection

### In-depth interviews

All the in-depth interviews will be directed at a place convenient for the pregnant women probably at a suitable place at the antenatal clinic. The PI will conduct the interviews, and a trained research assistant will be employed to get special notes where necessary during the interviews and record the interview. All interviews will be recorded with voice recorders, and the duration of an interview will be 30-45 minutes. Notes and recordings will be taken only with the participant’s consent. Even though the interviews will primarily focus on the interviewer guide, both primary and probing questions will be adjusted according to the study’s scope. An in-depth interviewer guide was prepared according to standard techniques. ^41^

### Focus group discussions

For the FGDs with PHMM, a focus group guide will be prepared, and interviews will be moderated according to standard techniques. The questions will be directed to explore the PHM’s observations on the reasons and implications of OOPE in pregnant women in her field practice area. All the interviews will be tape-recorded with the permission from PHMM, and the duration will be 45-60 minutes.

The interviewer guides will be pre-tested, and amendments will be made before the proper study.

### Data management

Interview notes will be expanded immediately after the interviews. All the discussions will be transcribed into MS Word document within one month and stored in a soft and hard copy file in a password-protected computer and locked cupboard, respectively, for each MOH area in a place where only accessible for PI and other investigators.

### Plan for data analysis

Data on interview transcripts will be analyzed using thematic analysis. In both instances, independent coding will be performed by PI, and another investigator trained in qualitative research. The coding schemes will be developed with consensus. The thematic analysis steps will include initial coding, coding scheme development, complete coding, identifying sub-themes and themes, and identifying the associations between different themes. Triangulation of data from pregnant women and PHMs will be performed to understand the social forces and implications of OOPE among free healthcare users. Data will be presented using appropriate visualization techniques and verbatims as evidence to elaborate information under each theme.

## Quality assurance of qualitative data

### Streamlining the data collection methods, tools, and analysis

The data collection tools and methods will be prepared in line with the accepted guidelines. The interviewer guide and other note-taker forms will be organized early. Interviewer guides will be pre-tested and the interviewer will use checklists to ensure data collection quality according to the Family Health International guidelines. ^41^ All the interviews will be done by PI. Transparency and rigor of the procedures will be considered with the data analysis methods. Two investigators knowledgeable on the concept of OOPE in free health systems and trained in qualitative analysis and will contribute in data analysis stage. Discussion among all the investigators will be done until consensus is reached when different opinions arise.

### Triangulation

We will collect data from two different individuals – pregnant women and PHMs – as collecting information from diverse individuals minimizes biases.

### Respondent validation

The interviewer will interpret the reasons for OOPE that have been revealed during the interviews and ask whether the participants would agree with such reasons at the end of the in-depth interviews and FGDs.

### Reflexivity

When conducting the interviews, one excepted bias is whether the participants may change or hide their actual expenditure details/reasons for OOPEs. Therefore, participants will be informed before the interview to avoid adopting behaviors and to provide true information to overcome this bias. Also, interviewers will assure that their pregnancy-related expenditures will not be commented on and judged on but only used for study purpose.

Another bias is in interpreting data in specific communities by the interviewers. It is doubtful whether the prior knowledge of investigators in different communities may impede during the interviews and in documentation. Therefore, the interviewers will maintain diaries and take memos during the investigation with the anticipation of considering them in the discussion of results and findings.

## Component 3: Finding the effects of COVID-19 outbreak on the household economy of pregnant women

### Study sample/participants

All pregnant women registered in the RaPCo study (n=3400) were invited to participate and pregnant women who were delivered at the period of the interview were eliminated from this section. The participation was optional due to the ongoing COVID-19 outbreak, and a sub-sample (n=1460) of RaPCo willingly attended the study.

### Data collection

Through telephone interviews, data collection was done using a pre-tested interviewer guide with close-ended questions from April to June 2020. Before the interview, participating pregnant women were informed of the study’s aims, and informed verbal consent to carry out the interview was taken. Interviews were conducted by six pre-intern medical officers who trained for data collection in telephone interviews. One interview was taken approximately 10 minutes and only focused on collecting concentrated information on the subject matter. Interviews were done only in two rounds. In the second round, all pregnant women who did not respond in the first round were interviewed. However, data collection was limited to two rounds only. Data were collected to obtain the following information during the COVID-19 outbreak.

• Status of utilizing maternal health services
• The assistance of the Public Health Midwife (PHM)
• The assistance of household members/neighbors
• Income loss during the outbreak
• Financial assistance

### Data management

All answers during the telephone interviews were entered into an MS Excel datasheet. Data cleaning was done and will be transferred into SPSS software for analysis. This component’s database was combined with the baseline database to identify the corresponding maternal health services and economic aspects’ related changes. The databases will be stored in a password-protected computer and the access will be limited only to the investigators.

### Plan for data analysis

Data will be analyzed with descriptive statistical measures; mean (SD), median (IQR), and frequencies. Parametric or non-parametric procedures (after checking normality) will be performed to identify the statistical significance.

In this study, the primary outcome variables are maternal service utilization and household income loss during the COVID-19 outbreak. Any change in maternal service utilization will analyze with descriptive analysis methods. The income changes will be explored compared to the previous income-related data gathered from the baseline survey of RaPCo.

## Ethical consideration

This study is a part of the RaPCo and the ethical approval for the RaPCo has been obtained from the ethics review committee of Faculty of Medicine and Allied Sciences, Rajarata University of Sri Lanka (ERC 2019/07).

## Confidentiality

Participants were assigned an ID number and used it throughout the study to maintain confidentiality. All consent forms were stored separately in our institution’s (Maternal and Child Health Research Unit [MCHRU]) locked filing cabinet. All questionnaires and transcribed documents were also stored in MCHRU and the access will be restricted only for the investigators.

## Dissemination of findings

All the study findings will be communicated to the scientific community as peer-reviewed research papers, abstracts, and presentations.

## Study status

The total recruitment of the RaPCo study was 3,400 pregnant women from July to October 2019, and 1,393 and 1,460 pregnant women willingly participated in the first and third components of this study. Up to the data management status, the research has been completed for the first and third components, and analysis will be done according to the proposed methods. In terms of study tool preparation, the second component was completed, and data collection will be started after pre-testing the study tool.

## Discussion

According to the global evidence, reducing the OOPE is a vital concern for increasing the health care demand for building a healthy nation. The impacts of productivity loss/cost are highly acknowledged by indicating the importance of absenteeism and presenteeism while at work or in household activities. However, there is a lack of evidence for both OOPE and productivity loss/cost regarding maternal health in Sri Lanka. Further, the impact of the ongoing crisis-the COVID-19 outbreak is questionable due to the lack of empirical evidence regarding maternal health. The proposed study will assess the OOPE, productivity loss/cost, and associated factors of OOPE, impacts, and causes of OOPE during pregnancy, and has implications of COVID-19 outbreak on pregnant women’s health service utilization and household economy Sri Lanka. This study collated evidence that would be valuable for policymakers, health care administrators, and health care practitioners globally, regionally, and locally.

Using a mixed-method approach such as quantitative and qualitative techniques on OOPE is essential to explore the magnitude, reasons, and impact of the OOPE among pregnant women to further improve the free maternal health services and reduce the household’s economic burden. Additionally, using a large sample for each component of the study covering all MOH areas in the district and data on associated factors will also help generate precise evidence. That would support planning further measures for reducing the OOPE, productivity loss/cost, and minimizing the hardship of the COVID-19 outbreak.

## Data availability

### Underlying data

No data are associated with this article.

### Extended data

Open Science Framework: Economic cost study tools. https://doi.org/10.17605/OSF.IO/GU2ED. ^42^

This project includes the following extended data:

- Baseline Survey – (Economic Cost Study) Interviewer-administred questionnaire
- Follow-up Survey – First Trimester (Economic Cost Study) Self-administered questionnaire
- Follow-up Survey – Second and Third Trimester (Economic Cost Study) Monthly basis self-administered questionnaire
- Hospital Survey – (Economic Cost Study) Interviewer-administred questionnaire

Data are available under the terms of the Creative Commons Attribution 4.0 International Public License.
