# Supplementary material for: Economic burden of out-of-pocket expenditure, productivity cost during pregnancy and COVID-19 impact on household economy in a cohort of pregnant women in Anuradhapura District, Sri Lanka; A study protocol
Source: F1000Res. 2022 Mar 4;10:700. Originally published 2021 Jul 30. [Version 3] doi: 10.12688/f1000research.53320.3 (PMC9046902; doi:10.12688/f1000research.53320.3)
Supplement: Peer review history

# Peer review: Reviewer response for version 3

**DOI:** 10.5256/f1000research.122081.r128667

**Published:** 2022-04-27

**Authors:** Francesco Salustri

**Affiliations:** 1 Institute for Global Health, University College London, London, UK

This article describes a study protocol that aims at investigating how economic costs, as measured by out-of-pocket expenditures (OOPE) and productivity costs, and COVID-19 impact pregnant women's household economy in Sri Lanka. More specifically, the study consists of three components: an analysis of the magnitude and the associated factors of the economic burden of OOPE and productivity costs, a qualitative case study to explore the role of free health services, and analysis on the role of COVID-19. For the first and the third components, the authors use a cohort of approximately 1,500 participants each, while the qualitative case study is assessed on 25 women.

 The protocol is very well described and the methodology combining both qualitative and quantitative analysis perfectly suits the aim of the research. The design is properly developed to answer all the research questions. The background is also well explained and highlights the relevance of the study for policymakers.

Is the study design appropriate for the research question?

Yes

Is the rationale for, and objectives of, the study clearly described?

Yes

Are sufficient details of the methods provided to allow replication by others?

Yes

Are the datasets clearly presented in a useable and accessible format?

Yes

Reviewer Expertise:

Health economics, microeconometrics.

I confirm that I have read this submission and believe that I have an appropriate level of expertise to confirm that it is of an acceptable scientific standard.

# Peer review: Reviewer response for version 3

**DOI:** 10.5256/f1000research.122081.r126319

**Published:** 2022-03-14

**Authors:** Ishanka Ayeshwari Talagala

**Affiliations:** 1 Disaster Preparedness and Response Division, Ministry of Health, Colombo, Sri Lanka; 2 Department of Community Medicine and Family Medicine, Faculty of Medicine, University of Moratuwa, Colombo, Sri Lanka

No further comments to be made.

Is the study design appropriate for the research question?

Yes

Is the rationale for, and objectives of, the study clearly described?

Yes

Are sufficient details of the methods provided to allow replication by others?

Yes

Are the datasets clearly presented in a useable and accessible format?

Not applicable

Reviewer Expertise:

Non communicable diseases, health system research, disaster management

I confirm that I have read this submission and believe that I have an appropriate level of expertise to confirm that it is of an acceptable scientific standard.

# Peer review: Reviewer response for version 2

**DOI:** 10.5256/f1000research.120116.r121495

**Published:** 2022-02-11

**Authors:** Ishanka Ayeshwari Talagala

**Affiliations:** 1 Disaster Preparedness and Response Division, Ministry of Health, Colombo, Sri Lanka; 2 Department of Community Medicine and Family Medicine, Faculty of Medicine, University of Moratuwa, Colombo, Sri Lanka

Thank you for the resubmission with the correction.

 However, in Methods, Component I: Study sample/participants:

 The authors have mentioned that only 40% of the RaPCo has volunteered for the study, and you were requested to provide evidence of generalizability within different socio-economic strata.

 Please note that the added paragraph does not serve that purpose.

 Also, you have mentioned, "There is no statistically significant difference among two groups of pregnant women in geographical areas....." However, the given statistics indicate otherwise.

Is the study design appropriate for the research question?

Yes

Is the rationale for, and objectives of, the study clearly described?

Yes

Are sufficient details of the methods provided to allow replication by others?

Yes

Are the datasets clearly presented in a useable and accessible format?

Not applicable

Reviewer Expertise:

Non communicable diseases, health system research, disaster management

I confirm that I have read this submission and believe that I have an appropriate level of expertise to confirm that it is of an acceptable scientific standard, however I have significant reservations, as outlined above.

# Response

**Published:** 2022-03-03

**Authors:** Sajaan Gunarathne

**Affiliations:** Rajarata University of Sri Lanka, Sri Lanka

Thank you very much for the valuable comment, and we agree with the reviewer. Thus, we used Chi-square goodness-of-fit test to check whether the proportions of several relevant variables of the economic study sample matches with the RaPCo sample, namely, religion, ethnicity, education level, and status of reproductive health education. The analysis showed no statistical significance differences (p>0.05). We have included the following edited paragraph in the revised manuscript.

 Since approximately 46% of the pregnant women in the RaPCo have participated in this study, we tested whether selected characteristics of the economic study sample (n=1573) differs those from the RaPCo (n=3367) sample using the Chi-square goodness-of-fit test. The analysis showed that there are no statistically significant differences in relation to religion [ *x* *2* *(df=1)=0.837, p=0.360* ], ethnicity [ *x* *2* *(df=1)=0.443, p=0.505],*  education level [ *x* *2* *(df=2)=3.919, p=0.141]*  and the status of sexual and reproductive health education [ *x* *2* *(df=1)=0.116, p=0.734]* .

# Peer review: Reviewer response for version 1

**DOI:** 10.5256/f1000research.56689.r101264

**Published:** 2021-12-13

**Authors:** Ishanka Ayeshwari Talagala

**Affiliations:** 1 Disaster Preparedness and Response Division, Ministry of Health, Colombo, Sri Lanka; 2 Department of Community Medicine and Family Medicine, Faculty of Medicine, University of Moratuwa, Colombo, Sri Lanka

Component 1:

1. The sample you are planning to take for the study is around 41% (1393 from estimated 3400 pregnant women). Please explain how you would maintain the generalizability within different socio-economic strata, especially since those who volunteer will be included in the study. Generally those who have comparatively higher level of education and those with high health seeking behaviour would volunteer for the study.
2. You have already used self-administered questionnaires for the follow up studies. Justify the use of self-administered questionnaire considering the educational and literacy level of the pregnant mothers in the district.

Component 2:

1. Please provide the potential variables that the interviewer guide for the in-depth interviews will include and then the guide for the focus group discussions.

Is the study design appropriate for the research question?

Yes

Is the rationale for, and objectives of, the study clearly described?

Yes

Are sufficient details of the methods provided to allow replication by others?

Yes

Are the datasets clearly presented in a useable and accessible format?

Not applicable

Reviewer Expertise:

not applicable

I confirm that I have read this submission and believe that I have an appropriate level of expertise to confirm that it is of an acceptable scientific standard, however I have significant reservations, as outlined above.

# Response

**Published:** 2022-01-07

**Authors:** Sajaan Gunarathne

**Affiliations:** Rajarata University of Sri Lanka, Sri Lanka

**Response to the Review Comments of the Manuscript – ‘Economic burden of out-of-pocket expenditure, productivity cost during pregnancy and COVID-19 impact on household economy in a cohort of pregnant women in Anuradhapura District, Sri Lanka; A study protocol’**

 Thank you for sending the reviewers' comments on the above manuscript and we have addressed all the comments provided by the reviewer and our response on all the comments are indicated below.

**Response to Reviewer:**

 Thank you for your valuable comments and suggestions on the manuscript. We have responded to each of your comments below.

**Component 1:**

**1) Comment** - The sample you are planning to take for the study is around 41% (1393 from estimated 3400 pregnant women). Please explain how you would maintain the generalizability within different socio-economic strata, especially since those who volunteer will be included in the study. Generally those who have comparatively higher level of education and those with high health seeking behavior would volunteer for the study.

**Response** – Thank you very much for the valuable comment. We understand that since approximately 40% of the original Rajarata Pregnancy Cohort (RaPCo) has volunteered for this study, there is an issue with the generalizability of the study findings.

 However, we have socio-demographic data of all the pregnant women of the RaPCo. We used the Chi-square test to see whether the economic study sample (n=1393) matches the socio-demographic data of the RaPCo (n=3400).

 There is no statistically significant difference among two groups of pregnant women in geographical areas (22 MOH areas in the Anuradhapura District) [ *x* *2* *(df=21)=90.83, p=0.0001]* , education level of the pregnant women [ *x* *2* *(df=4)=17.76, p=0.003]*  and the status of sexual and reproductive health education of pregnant women [ *x* *2* *(df=1)=6.53, p=0.038]* .

 This was also included in the manuscript under the study sample/participants in the first components of the study.

**2) Comment** - You have already used self-administered questionnaires for the follow up studies. Justify the use of self-administered questionnaire considering the educational and literacy level of the pregnant mothers in the district.

**Response – **Thank you very much for the comment. We understand that using a self-administered questionnaire in a rural setting is challenging. Therefore, we used the following measures to avoid the incompleteness of filled questionnaires.

 When distributing the questionnaires, all the guidelines to fill the questionnaires were given by the principal investigator or another research team member. We asked pregnant women to fill the questionnaire with the help of a family member if the pregnant women are less aware of the household economy and health expenditure-related information. 

 In addition, an information leaflet was attached to the self-administered questionnaire, and it included all the guidelines to fill the questionnaires.

 Further, to minimize loss to follow-up, telephone reminders were given monthly to all pregnant women and reminded them to fill out the questionnaires and helped if any issue occurred. 

**Component 2:**

**1) Comment** - Please provide the potential variables that the interviewer guide for the in-depth interviews will include and then the guide for the focus group discussions.

**Response** – Thank you very much. The interviewer guides for in-depth interviews and focus group discussion guide was added to the manuscript under the ‘extended data’.

 Further, the following sentences were included in the manuscript under the titles of ‘In-depth interviews’ and ‘Focus group discussions’, respectively.

 Under the title ‘In-depth Interviews’ – “An in-depth interviewer guide was prepared according to standard techniques and focused on the socio-economic background of pregnant women, information on preferable health service, details of OOPE, reasons for OOPE and impact of OOPE.”

 Under the title ‘Focus Group Discussions’ – “The guide was focused on preferable health service, experience with OOPE of pregnant women, reasons for OOPE and impacts of OOPE in PHMs’ view.”

 Thank You

 Sajaan Praveena Gunarathne
